# Myopathy and ataxia related to impaired mitochondrial function in mevalonate kinase deficiency

**DOI:** 10.1186/s13023-026-04248-y

**Published:** 2026-02-12

**Authors:** Alessia Pugliese, Christina von Landenberg, Romina Gallizzi, Alba Migliorato, Sabata Pierno, Carmelo Rodolico, Cornelia Kornblum, Ignazio Giuseppe Arena, Wolfram S. Kunz, Jens Reimann, Antonio Toscano, Olimpia Musumeci

**Affiliations:** 1 Department of Clinical and Experimental Medicine, University of Messina, Messina, Italy; 2 Department of Neurology, University Hospital Bonn, Bonn, Germany; 3 BioNTech SE, Mainz, Germany; 4 Department of Medical of Health Sciences, Magna Graecia University, Catanzaro, Italy; 5 Biomorphology, Dental Sciences and Morphological and Functional Images, University of Messina, Messina, Italy; 6 Section of Pharmacology, Department of Pharmacy - Drug Sciences, University of Bari Aldo Moro, 70125 Bari, Italy; 7 Center for Neurology, Department of Neuromuscular Diseases, University Hospital Bonn, Bonn, Germany; 8 Life & Brain Center, Department of Epileptology, University Hospital Bonn, Bonn, Germany

**Keywords:** Mevalonate kinase deficiency, Mevalonic aciduria, Coenzyme Q10, Mitochondrial dysfunction, Ataxia, Metabolic myopathy, hyperCKemia

## Abstract

**Background:**

Mevalonate kinase deficiency (MKD) is a rare genetic disorder, resulting in the lack of the mevalonate kinase enzyme (MVK), which is involved in the biosynthesis of cholesterol, non-sterol isoprenoids, and coenzyme Q10 (CoQ10). The more severe phenotype of MKD is known as mevalonic aciduria (MA), typically presenting as a multisystemic inflammatory syndrome with possible neurological manifestations, such as developmental delay, cerebellar ataxia, and retinopathy. Myopathy or isolated hyperCKemia have been rarely reported in association with MA. However, a few studies evidenced mitochondrial dysfunction in MVK deficient cells.

**Aim:**

To point out the connection between MKD, myopathy, and mitochondrial dysfunction, describing two cases of MA.

**Methods:**

We report on two unrelated patients with myopathy and ataxia, providing clinical, histological, biochemical, and genetic data of MKD.

**Results:**

Both patients were referred to the Neurology Department in the first year of life, due to muscle weakness, gait disturbances, and increased levels of CK value. Muscle biopsy was performed, showing some mitochondrial alterations and mild lipid storage. Interestingly, biochemical studies on muscle homogenate revealed a reduction of mitochondrial respiratory chain activities and CoQ10 levels. Genetic analysis confirmed the MKD diagnosis, evidencing a homozygous *MVK* gene mutation in the first case, and compound heterozygous mutations in the second one.

**Conclusion:**

This report describes two MKD cases with clinical and morphological evidence of muscle involvement in the spectrum of MA related to mitochondrial dysfunction.

## Introduction

Mevalonate kinase deficiency (MKD) is a rare inborn error of isoprenoid biosynthesis, presenting as a multiorgan autoinflammatory disease. MKD is currently considered as a broad spectrum of phenotypes, identifying the two extremes in a milder form, called hyperimmunoglobulinemia D and periodic fever syndrome (HIDS, OMIM#260920), and a more severe and rarer one, known as mevalonic aciduria (MA, OMIM#610377). Both disorders are characterized by recurrent inflammatory attacks, with abrupt onset of fever, arthralgia, myalgia, skin rush, gastrointestinal symptoms, and mucosal ulcers. Additionally, patients affected by MA may suffer from developmental delay, dysmorphism, progressive cerebellar ataxia, and hypotonia [1, 2].

The epidemiology of MKD is highly variable among the different countries, with an estimated prevalence of 300 people worldwide. A prevalence peak has been registered in the Netherlands, probably due to a Dutch founder effect [3, 4].

MKD is caused by biallelic variants in mevalonate kinase gene (*MVK*), located on chromosome 12q24 and encoding for the homonym protein MVK, the key enzyme of mevalonate pathway. Missense mutations have been more frequently reported, resulting in a just partial enzyme deficiency. Indeed, the complete absence of the enzyme would result in intrauterine lethality [5].

**Fig. 1:**
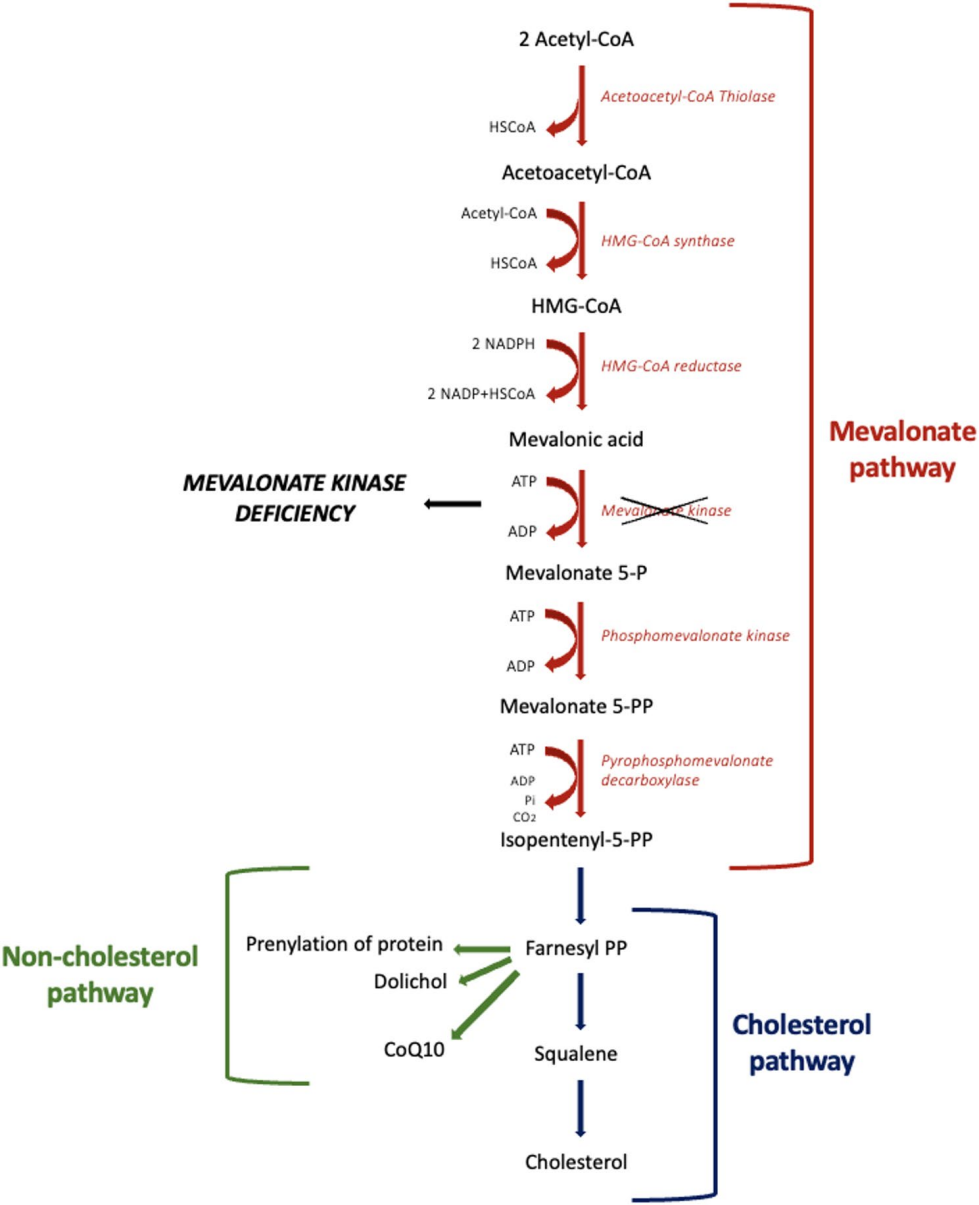
Overview of the mevalonate pathway. Mevalonate pathway leads to the synthesis of Isopentenyl-5-PP, which is next converted into Farnesyl-PP. Farnesyl-PP starts the cholesterol and non-cholesterol pathways, resulting in resulting in the production of cholesterol, dolichol, CoQ10, and metabolites responsible for several proteins prenylation. In MKD, a blockade occurs at the conversion of mevalonic acid into mevalonate 5-P, due to deficiency of mevalonate kinase enzyme. Abbreviations: HSCoA Coenzyme A; HMG-CoA 3-hydroxy-3-methylglutaryl-coenzyme A; NADPH Nicotinamide adenine dinucleotide phosphate hydrogen; NADP Nicotinamide adenine dinucleotide phosphate; P Phosphate; PP Pyrophosphate; CoQ10 Coenzyme Q10; MKD mevalonate kinase deficiency

The pathogenesis of the disease is still not completely understood. MVK is involved in the synthesis of cholesterol and its branched unsaturated lipid chains called non-sterol isoprenoids (Fig. 1). Reduced levels of isoprenoids lead to increased secretion of interleukin 1b (IL-1b) and hence hyperinflammation. Therefore, *MVK* mutations determine the accumulation of the upstream mevalonic acid, excreted in the urine of the affected patients. Furthermore, the production of metabolites that are crucial for cellular respiratory function is disrupted, with subsequent mitochondrial damage, incomplete autophagy, and cell death [4].

Neurological symptoms, such as intellectual disability, developmental delay, ataxia, and retinal degeneration, have been sometimes described as part of MKD clinical picture. In the last years increasing attention has been paid to the possible mechanisms underlying the development of MKD neurological manifestations [6–8]. Indeed, MVK is an enzyme shared between the synthesis pathways of cholesterol and Coenzyme Q10 (CoQ10), whose deficiency is counted among the causes of cerebellar ataxia. Since CoQ10 also plays a crucial role in the mitochondrial respiratory chain (RC), CoQ10 deficiency could contribute to the pathogenesis of MKD [9].

We describe herein two unrelated patients affected by MA, exhibiting the typical inflammatory phenotype with myopathy and ataxia, related to RC and CoQ10 deficiency.

## Patients and methods

We report two unrelated cases of Italian and German origin. Both patients were referred to the Neurology Department for a clinical suspicion of neuromuscular disease, due to muscle weakness, gait disturbances, and increased serum CK levels. At the time of his neurological referral, the first patient had already received MKD diagnosis at the Pediatric Department. In detail, case 1 was diagnosed and followed up at the University Hospital of Messina, Italy, while case 2 was firstly evaluated and diagnosed at the Neurology Department of the University Hospital of Bonn, Germany.

To deeply investigate the nature of muscle involvement, both patients performed an open muscle biopsy on vastus lateralis muscle, including histochemical and biochemical assays on muscle homogenate.

### Case 1

The first patient is an Italian boy, aged 20 years at the last follow-up. He was first-born to consanguineous parents (first cousins’ marriage), after an uneventful pregnancy and cesarean delivery. Family history was positive for neurological and rheumatological diseases, in particular his mother was affected by multiple sclerosis and familial mediterranean fever. Five months after birth, the baby suffered from recurrent febrile attacks with erythema, lymphadenomegaly, and diarrhea. An extensive newborn screening was performed at the Pediatric Department of the University Hospital of Messina, Italy. High levels of mevalonic acid were detected in the patient’s urine, suggesting the diagnosis of MKD. The disease was later confirmed by genetic analysis. Therefore, a therapy with multivitamin supplements and anti-inflammatory medications was started. Around the 9th month of life, diffuse joint tumefactions appeared and non-responder microcytic anemia was diagnosed. Moreover, his weight was 2–4 SD (standard deviation) below the mean, and he required naso-gastric tube to deal with malnutrition till the age of 10 years. When he was 2 years old, he started therapy with Anakinra, an anti IL1-receptor (interleukin 1) treatment, that was partially effective in remitting febrile episodes. For the persistence of arthralgia, therapy with Etanercept, an anti-TNFα agent (tumor necrosis factor α), was later performed in addition with cyclosporine, methotrexate, and steroids. Since the age of 8 years, he was treated with a disease specific monoclonal antibody therapy targeting IL-1β (canakinumab), with mild benefit. Unfortunately, both steroid treatment and inflammatory condition caused diffuse osteoporosis, requiring treatment with calcium, vitamin D, and neridronato. Finally, he took also acetylsalicylic acid for high platelets plasma levels.

The boy was referred to the Neuromuscular Department at the age of 12 years, after the worsening of gait disturbances and detection of mildly increased serum creatine kinase (CK) levels (600–1900 IU/l; n.v. 0-200). The first neurological examination revealed dysmorphisms (high arched palate, low set ears, micrognathia, pectus carinatum, scoliosis, kyphosis, and flat feet), ataxic gait, several joint contractures, distal tremor at upper limbs, distal muscles weakness at four limbs (Medical Research Council strength scale – MRC grade 4) and hyperreflexia, in addition to mild delay in language learning at cognitive evaluation. Electromyography (EMG) showed normal conduction nerve velocities and reduced duration and amplitude of polyphasic motor unit potentials with an early recruitment pattern under maximal exertion, on both upper and lower muscles. Brain MRI (magnetic resonance imaging) evidenced a diffuse cerebellar atrophy.

**Fig. 2:**
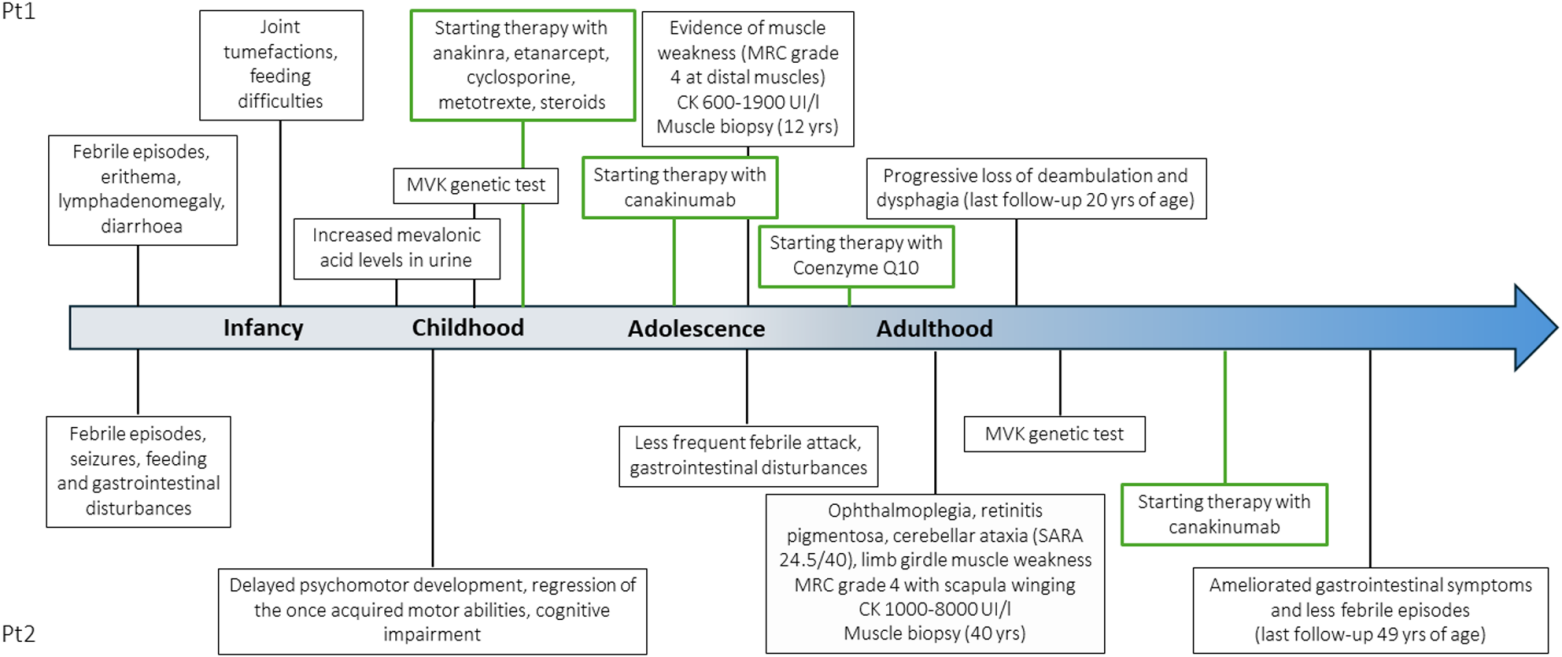
Clinical, diagnostic and therapeutic timeline of the patients. A representation of clinical manifestations, diagnostic tools and therapies (highlighted in green) of the two patients along the course of the disease

Over the years, febrile attacks and cutaneous rash disappeared, while muscular involvement became prevalent (Fig. 2).

### Case 2

The second patient is a 49-year-old man, born to German non-consanguineous parents. He presented at the Neurology Department of the University Hospital of Bonn, Germany, outpatient clinic as a suspected mitochondrial disorder. The first evidence of a potential multisystem disease occurred at an early postnatal age, when the patient presented with feeding problems, unexplained febrile episodes, seizures, and gastrointestinal disturbances (i.e. abdominal pain and diarrhea). Neurological exam in adulthood showed ophthalmoplegia, marked visual impairment due to retinitis pigmentosa, cerebellar ataxia with a SARA score (scale for the assessment and rating of ataxia) of 24.5/40, and limb girdle muscle weakness MRC grade 4 with scapula winging. Psychomotor development was delayed markedly, in association with a regression of the once acquired motor abilities, and cognitive impairment was found. Serum CK levels were elevated (1,000–8,000 IU/l; ULN 190). At an adult age, decline of motor functions decelerated and seizures did not reoccur. Although duration and frequency of febrile attacks decreased over time, those episodes still occurred several times per year, occasionally requiring hospitalization. Furthermore, the patient continuously suffered from frequent gastrointestinal disturbances, which only became apparent after consulting the patient’s mother and keeping a symptom diary. Taken together, the patient’s persisting signs of disease activity led us to initiate canakinumab, applied according to the recommendations of the Summary of Product Characteristics (SmPC). Under this therapy the patient’s gastrointestinal symptoms ameliorated considerably and febrile episodes became less frequent, as confirmed by regular three-monthly follow-up examinations, continued symptom diaries and reports by the patient’s mother (Fig. 2).

It is worth mentioning that the younger brother of the patient was affected by the same disorder, presenting with less severe ataxia, visual field defects and marked visual loss.

Some clinical data of case 2 and his brother have been already published [6]. 

### Methods

#### Muscle biopsy and biochemical studies

10-µm-thick frozen cross sections were processed for histological (Hematoxylin & Eosin -H&E- and Gomori trichrome) and histochemical (cytochrome oxidase -COX- and succinate dehydrogenase -SDH-) studies, according to standard techniques. Biochemical analyses, including complex activity measurement and total CoQ10 quantification on homogenate from frozen muscle, were carried as previously described [10–12]. Specifically, NADH dehydrogenase (complex I), rotenone-sensitive NADH-cytochrome c reductase (complex I - III), succinate-cytochrome c reductase (complex II - III), succinate dehydrogenase (complex II), cytochrome c oxidase (complex IV), and citrate synthase were assessed spectrophotometrically. RC enzyme activities were referred to the activity of citrate synthase [10]. Total CoQ10 concentration was measured using a modification of the method described by Lang et al. CoQ10 extraction was performed at 4 °C with ethanol-n-hexane. After complete evaporation under a stream of nitrogen, the residue was dissolved in 1.0 mL of ethanol/methanol mixture (65:35 volume to volume ratio) and the concentration of total CoQ10 was determined by reverse phase high-performance liquid chromatography (HPLC) on a C18 column and ultraviolet detection at 275 nm, using CoQ9 as an internal standard [11, 12].

#### Genetic analysis

In the first patient, *MVK* gene was analysed with PCR amplification, sequencing the entire codifying region and the immediately flanking intronic regions, after the urine detection of high levels of mevalonic acid. In the second case, long range PCR and sequencing of mtDNA, RFLP (Restriction Fragment Length Polymorphism) analysis for variants in POLG gene, and analysis of genes causing spinocerebellar ataxia were firstly performed. Then, NGS (next generation sequencing) for genes causing retinitis pigmentosa, including *MVK* gene, was performed.

## Results

### Muscle biopsy and biochemical studies

In the first case, morphological studies showed about 10% of vacuolated fibres, with mild lipid accumulation at the Sudan Black staining. Biochemical studies on muscle specimen detected a reduction of complex I (6,7 nmol/min/mg prot; normal range (n.r.) 22.6 ± 6; complex I/citrase synthase activity (CS): 0.06; n.r. 0.16 ± 0.02, residual activity 43%) and complex III (4,3 nmol/min/mg prot; n.r 11.6 ± 2.3; complex III/CS: 0.04; n.r. 0.11 ± 0.01, residual activity 40%) activities. Total muscle CoQ10 was significantly decreased (14 µg /g wet muscle; n.v. 25 ± 4; 56%) [10]. According to CoQ9 values as internal control, the extraction recovery was about 87%.

**Fig. 3:**
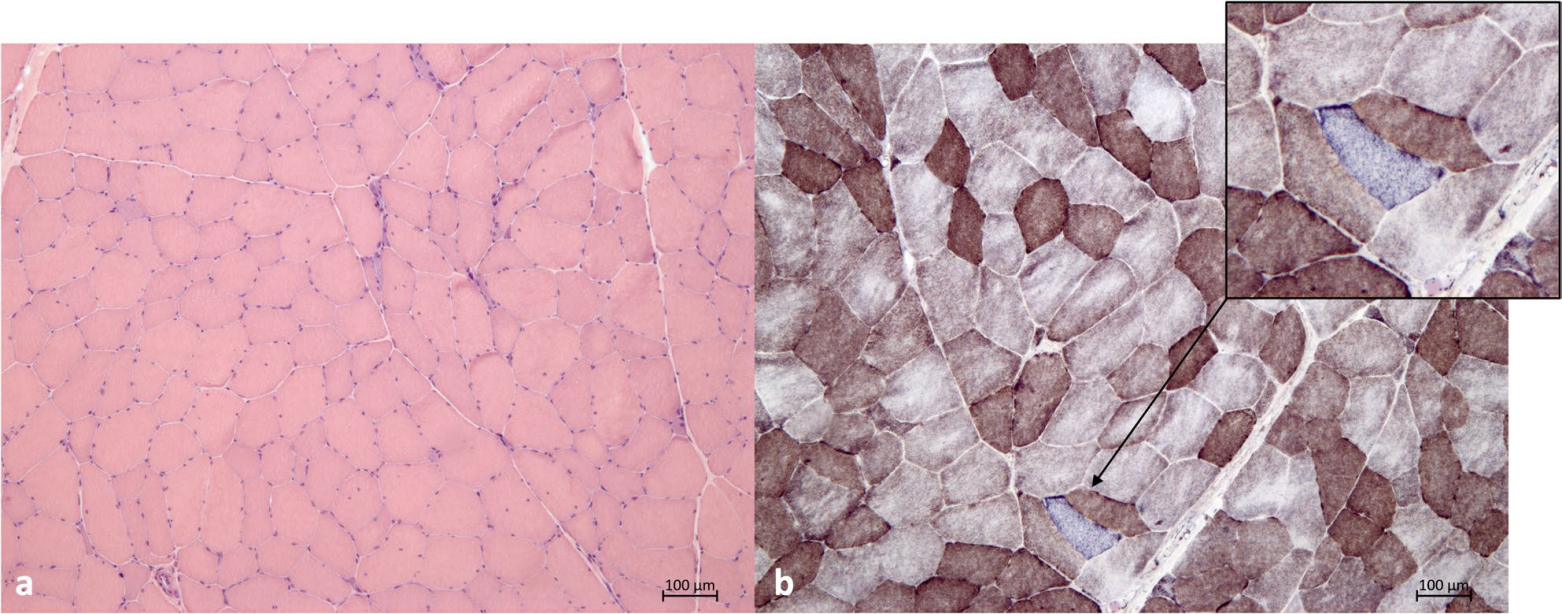
Muscle biopsy in patient 2. (**A**) H&E stain with increased fibre size variability showing atrophic and hypertrophic fibres as well as internalized myonuclei. Primary magnification x10. (**B**) Cytochrome c oxidase (COX) and succinate dehydrogenase (SDH) double enzyme histochemistry demonstrating irregular distribution of mitochondrial enzyme activities and a COX-negative, SDH-positive fibre (insert). Primary magnification x10

In the second case, muscle biopsy showed unspecific myopathic changes, consisting of increase of fibres size variation and centralized nuclei about 20%, sporadic detection of ghost fibres and myophagia, as well as some small dark angulated fibres. Histological signs of putative mitochondrial disorder were few scattered COX-negative/SDH-positive fibers (0.8%) and abnormal mitochondrial distribution (Fig. 3). RC activity measurement revealed a marked reduction of complex IV (1.03 U/ g wet weight; n.r. 9.41 ± 2.87; complex IV/CS: 0.1; n.r. 0.68 ± 0.15- residual activity 19%). Muscle CoQ10 concentration was also decreased (11.7 µg /g wet muscle; n.v. 25 ± 4; 46%).

### Genetic analysis

In the first patient, *MVK* sequencing revealed the variant c.60T > A (p.His20Gln) in a homozygous state. This variant is classified as likely pathogenic according with ACMG criteria and was already reported in other MKD cases [5, 13].

In the second case, long range PCR and sequencing of mtDNA, RFLP (Restriction Fragment Length Polymorphism) analysis for variants in *POLG* gene, and analysis of genes causing spinocerebellar ataxia did not reveal a causative mutation. At the age of 43 years, NGS panel for genes causing retinitis pigmentosa, including *MVK* gene revealed compound heterozygous variants in the *MVK* [*c.59 A > C* (p.His20Pro) and *c.1000G > A* (p.Ala334Thr)]. Both variants have been classified as pathogenic by ACMG criteria and already reported in association with MVK deficiency [14–16]. Segregation analysis confirmed the heterozygous state in the patient’s parents.

### Clinical follow-up

After the identification of muscle CoQ10 deficiency, patient 1 started supplementation with CoQ10 (300 mg per day). He was annually evaluated with neurological examination focusing on motor function (MRC score) and SARA score. No evidence of improvement was detected over the years and, at the last examination, after 5 years of CoQ10 supplementation, the patient was wheelchair-bound. He showed progressive solid dysphagia. Joint contractures worsened, reducing the abduction and elevation of both arms and inducing bilateral elbow and knee flexion position. Limbs hypometria, diffuse muscle hypotrophy and upper limbs hypotonia were also evident, in addition to upper limb muscles weakness, mainly involving extensor and flexor carpi muscles, extensor and flexor digitorum muscles, and intrinsic hand muscles. CK level remains elevated about 6-fold upper limit of normal range.

CoQ10 supplementation has been continued till the last follow-up with apparent poor benefit (loss of deambulation) on muscle manifestations, despite patient’s good compliance. (Fig. 2).

## Discussion

MKD is a rare inherited disease, characterized by a highly heterogeneous clinical presentation. Recurrent fever episodes, abdominal pain, skin rash, and arthralgia represent the most frequent clinical manifestations in MA, in addition to psychomotor impairment, dysmorphic facial features, and cerebellar ataxia [17].

In this paper, we describe two unrelated Caucasian patients affected by MA, presenting marked neurological alterations beyond the typical MKD autoinflammation syndrome (Table 1). Cerebellar involvement mainly expressing with gait ataxia, and further supported by MRI cerebellar atrophy, was noticed in both patients. Besides, ophthalmoplegia and retinitis pigmentosa were evident in the case 2. A few MKD patients affected by a predominant neurological phenotype (i.e. intellectual disability, cerebellar atrophy, ataxia, hypotonia, and retinal dystrophy) with mild or no inflammation symptoms have been described in association to compound heterozygous mutations *c.59 A > C/c.1000G > A* [6, 16–18]. A recent study hypothesized a genotype-phenotype correlation between these variants and an isolated neurological presentation [8]. Our second patient harboured the same genetic alteration with predominant neurological phenotype. However, febrile attacks as well as recurring gastrointestinal disturbances were reported since postnatal age till the adulthood, although ameliorated under anti-IL-1β therapy.

**Table 1:** Clinical features and diagnostic findings

Patients	Onset	General clinical features	Neurological features	Serum CK levels	Brain MRI	EMG	Muscle biopsy	Biochemical assays	Genotype
**Case** 1 Male, 20 yrs	Early postnatal age	Recurrent febrile attacks, erythema, arthralgia, lymphadenomegaly, GI symptoms (i.e. diarrhea), microcytic anemia, thrombocytosis, Osteoporosis	Developmental delay, nystagmus, mild solid dysphagia, upper limbs hypotonia, diffuse muscle hypotrophy distal upper limb muscles weakness	600-1,900 IU/l	Diffuse cerebellar atrophy	Myopathic pattern	Some vacuolated fibres with mild lipid storage (10%)	*Complex I/CS: 43% *Complex III/CS: 40% CoQ10: 14 µg/g wet muscle (n.v. 25 ± 4)	Homozygous for c.60T > A (p.His20Gln)
**Case** 2 Male, 49 yrs	Early postnatal age	Febrile attacks, feeding problems, GI symptoms (i.e. abdominal pain, diarrhea)	Developmental delay, seizures, ophthalmoplegia, retinitis pigmentosa, cerebellar ataxia, limb girdle muscle weakness	1,000–8,000 IU/l	Mild cerebellar atrophy	Mild myopathic changes	Increased fibre size variation, a centralized nuclei (20%), sporadic ghost fibres, myophagia, few COX-negative/SDH-positive fibres abnormal mitochondrial distribution	*Complex I: normal *Complex IV/CS: 19% CoQ10: 11.7 µg/g wet muscle (n.v. 25 ± 4)	Compound heterozygous for c.59 A > C/c.1000G > A (p.His20Pro/p.Ala334Thr)

*Mitochondrial respiratory chain activities are reported as percentage of residual activity in comparison with the normal value, normalized to citrate synthase (CS) (%)

Abbreviations: GI gastrointestinal; COX cytochrome-c oxidase; SDH succinate dehydrogenase; CoQ10 Coenzyme Q10

Facial dysmorphic features are usually reported as part of the MKD phenotype, including frontal bossing, hypertelorism, low set ears, and triangular shape face. Apart from facial dysmorphism, patient 1 showed some skeletal deformities as upper limbs hypoplasia, pectus carinatum, scoliosis, kyphosis, and flat feet, that are not commonly reported. These alterations in addition with diffuse joint retractions at four limbs severely impaired the motor abilities of the patient (Fig. 2).

In our patients, muscle involvement was evident affecting distal upper limb muscles in case 1 and limb-girdle muscles in case 2. Muscle damage was indicated by elevated serum CK levels and myopathic pattern at EMG. Both patients underwent muscle biopsy, which confirmed myopathic alterations, such as increased fibres size variation, internalized nuclei, a few mitochondrial changes and some vacuolated fibres with lipid storage. Interestingly, both patients had a multiple complex RC deficiency, in fact patient one had a reduced complex I and III activity (residual activity 43% and 40% respectively), whereas patient two presented a marked complex IV activity reduction (residual activity 19%).

**Fig. 4:**
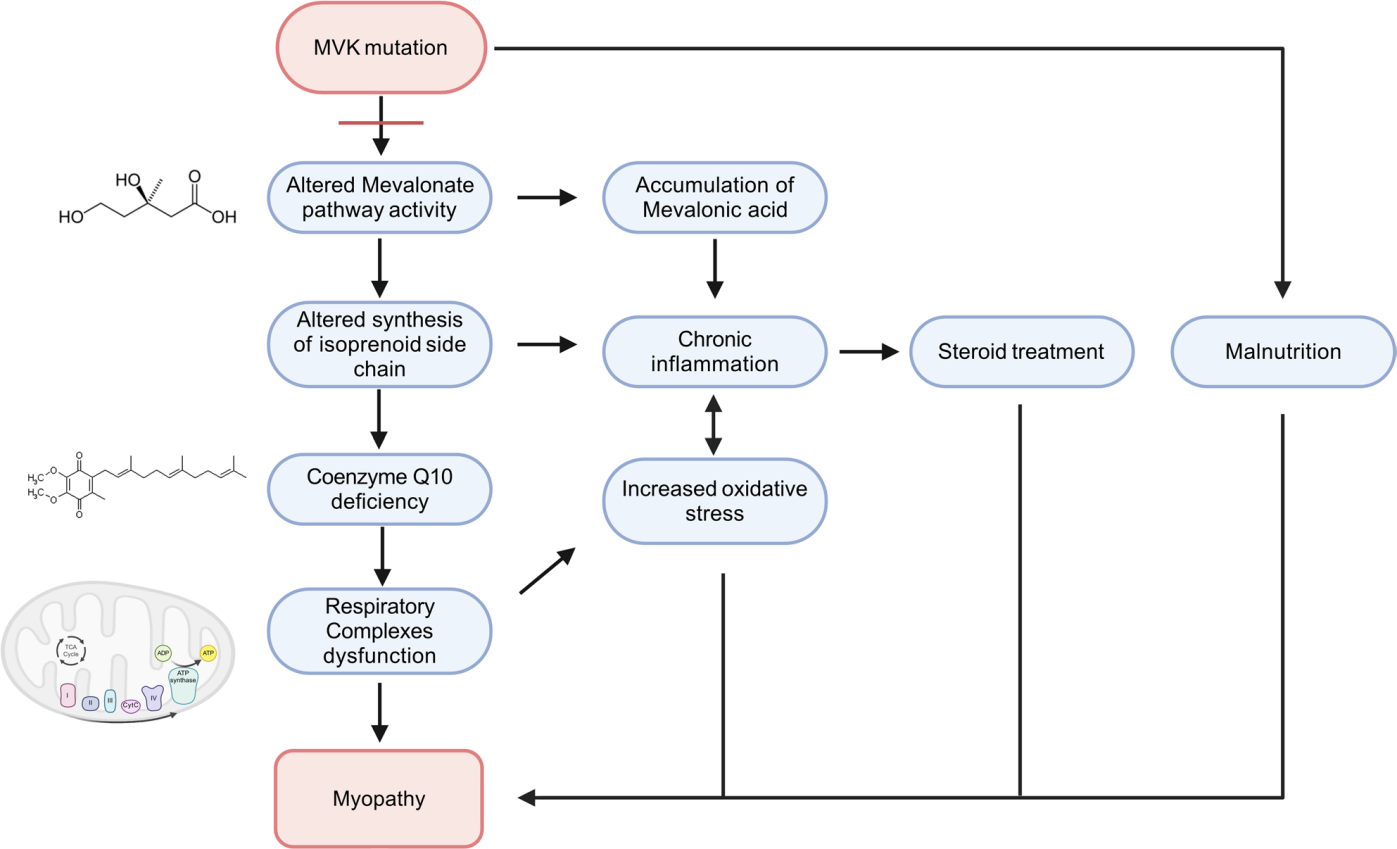
Schematic representation of impaired mevalonate pathway. The dysfunction of the mevalonic pathway can directly damage the muscle, although other factors may contribute to the damage (i.e. steroid treatments, malnutrition, and chronic inflammation)

Reduced RC activity is a result of altered mitochondrial functions, as already described for MKD [19]. MVK catalyses the mevalonic acid conversion to 5-phosphomevalonic acid, resulting, if deficient, in elevated plasmatic and urinary levels of mevalonic acid [20]. The pathogenic mechanism of muscle involvement in MKD is not well elucidated so far. The impairment of the mevalonic pathway can directly contribute to muscle damage, although it is not possible to exclude other factors as steroid treatments, malnutrition, and chronic inflammation (Fig. 4). Loss of MVK activity leads to both accumulation of mevalonic acid and deficiency of the downstream compounds, including cholesterol and non-sterol isoprenoids, and CoQ10 [3].

In line with the above considerations, both patients presented multiple complex RC deficiency and muscle CoQ10 levels reduction (56% and 46% than normal respectively). Recent studies on the effects of impaired mevalonate pathway in Saccharomyces cerevisiae demonstrated that both the shortage of isoprenoids and the depletion of CoQ10 could be responsible for RC dysfunctions in MVK-defective cells, triggering autophagy and specifically mitophagy [21, 22].

These findings suggest a link between MVK deficiency, inflammation, and mitochondrial alterations. Previous studies showed lower CoQ10 concentration in plasma and cultured fibroblasts, but data on muscle biopsy are rarely reported [19, 23, 24]. *Hübner C. et al.* speculated that, in nine out ten of reported MKD patient, low levels of CoQ10 could be responsible for an increased oxidative stress, accounting for the observed cerebellar ataxia and myopathy [24]. In other reports, CoQ10 levels in fibroblasts were quite normal.

Patient 1 was treated with 300 mg/day of CoQ10 but no evident improvement was noted according to the annual clinical evaluation over five years of follow up, pointing out other possible concomitant factors in the MKD pathology. Despite this, it has been suggested that CoQ10 supplementation could be useful in order to reduce intracellular reactive oxygen species and improve impaired mitochondrial energy metabolism [18].

## Conclusion

In conclusion, this report describes two MKD cases, focusing on myopathy as part of MKD phenotype probably due to a mitochondrial impairment and CoQ10 reduction. Conversely, MA should be excluded in patients affected by metabolic myopathy which do not fit the more common diagnosis, especially in presence of recurrent and unexplained febrile episodes, key indicators of inflammatory response in MKD.

It is likely that MA is underdiagnosed due to its highly variable presentation. However, taking into account a wider clinical spectrum for MKD could be helpful to promptly recognize this disorder.

## Data Availability

The data supporting the conclusions of this article are included within the article.

## Abbreviations

MKD: Mevalonate kinase deficiency
HIDS: Hyperimmunoglobulinemia D and periodic fever syndrome
MA: Mevalonic aciduria
*MVK*: Mevalonate kinase gene
MVK: Mevalonate kinase enzyme
CoQ10: Coenzyme Q10
RC: Respiratory chain
MRC: Medical research counsil
IL1: Interleukin 1
IL-1b: Interleukin 1b
TNFα: Tumor necrosis factor α
CK: Creatine kinase
COX: Cytocrome-c oxidase
SDH: Succinate dehydrogenase
mtDNA: Mitochondrial DNA
SARA score: Scale for the assessment and rating of ataxia
PCR: Polymerase chain reaction
RFLP: Restriction fragment length polymorphism
NGS: Next generation sequencing
MRI: Magnetic resonance imaging
ULN: Upper limit of normal
CS: Citrate synthase
